# The Effects of Taekwondo Training on Peripheral Neuroplasticity-Related Growth Factors, Cerebral Blood Flow Velocity, and Cognitive Functions in Healthy Children: A Randomized Controlled Trial

**DOI:** 10.3390/ijerph14050454

**Published:** 2017-04-25

**Authors:** Su-Youn Cho, Wi-Young So, Hee-Tae Roh

**Affiliations:** 1 Department of Taekwondo, Youngsan University, Yangsan-si 50510, Korea; csy@ysu.ac.kr; 2 Sports and Health Care Major, College of Humanities and Arts, Korea National University of Transportation, Chungju-si 27469, Korea; wowso@ut.ac.kr; 3 Department of Physical Education, College of Arts and Physical Education, Dong-A University, Busan 49315, Korea

**Keywords:** cerebral blood flow, children, cognition, neurotrophic factors, Taekwondo

## Abstract

Although regular Taekwondo (TKD) training has been reported to be effective for improving cognitive function in children, the mechanism underlying this improvement remains unclear. The purpose of the present study was to observe changes in neuroplasticity-related growth factors in the blood, assess cerebral blood flow velocity, and verify the resulting changes in children’s cognitive function after TKD training. Thirty healthy elementary school students were randomly assigned to control (*n* = 15) and TKD (*n* = 15) groups. The TKD training was conducted for 60 min at a rating of perceived exertion (RPE) of 11–15, 5 times per week, for 16 weeks. Brain-derived neurotrophic factor (BDNF), vascular endothelial growth factor (VEGF), and insulin-like growth factor-1 (IGF-1) levels were measured by blood sampling before and after the training, and the cerebral blood flow velocities (peak systolic [MCAs], end diastolic [MCAd], mean cerebral blood flow velocities [MCAm], and pulsatility index [PI]) of the middle cerebral artery (MCA) were measured using Doppler ultrasonography. For cognitive function assessment, Stroop Color and Word Tests (Word, Color, and Color-Word) were administered along with other measurements. The serum BDNF, VEGF, and IGF-1 levels and the Color-Word test scores among the sub-factors of the Stroop Color and Word Test scores were significantly higher in the TKD group after the intervention (*p* < 0.05). On the other hand, no statistically significant differences were found in any factors related to cerebral blood flow velocities, or in the Word test and Color test scores (*p* > 0.05). Thus, 16-week TKD training did not significantly affect cerebral blood flow velocities, but the training may have been effective in increasing children’s cognitive function by inducing an increase in the levels of neuroplasticity-related growth factors.

## 1. Introduction

The mechanisms by which exercise increases nerve cell production are not clearly understood, but an increase in nerve growth factors and neurotransmitters during exercise has been suggested to play an important role. Specifically, brain-derived neurotrophic factor (BDNF) is well known to show an exercise-induced increase in expression and promote neuronal cell formation [1]. BDNF is involved in promoting the survival of progenitor cells that have the potential to differentiate into neurons or glia, as well as in differentiating these progenitor cells into neurons [2]. Neurotransmitters, such as serotonin, also show increased levels after exercise and have been reported to induce the generation of neurons [3]. In addition, various growth factors, such as vascular endothelial growth factor (VEGF) and insulin-like growth factor 1 (IGF-1), are known to promote the production of new cells [4].

In addition, the development of image analysis techniques, such as functional magnetic resonance imaging (fMRI), positron emission tomography (PET), and transcranial Doppler (TCD), has not only facilitated morphometric analysis of the brain, but has also allowed accurate confirmation of the brain regions activated by various stimulations. Among these image analysis techniques, TCD is a non-invasive method that allows real-time measurement of intracranial hemodynamics without requiring exposure to radiation and contrast agents, but it cannot visualize whole blood vessels [5]. Previous studies have suggested that regular exercise can reduce the risk of cerebrovascular and neurological diseases by increasing cerebral blood flow. Murrell et al. reported that a 12-week aerobic exercise training program significantly increased middle cerebral artery blood velocity (MCAv) in young (mean age: 23 ± 5 years) and older (mean age: 63 ± 5 years) participants [6], while Ainslie et al. reported that regular aerobic-endurance exercises were significantly correlated with high MCAv in men aged 18–79 years [7]. Studies that have examined changes in MCAv with regular exercise in children and/or adolescents during their growth period are limited; previous studies in this regard were limited to specific conditions, such as vasovagal syncope [8,9].

Taekwondo (TKD) is a martial art form that originated in Korea and has been adopted as an official Olympic sport since the Sydney Olympics in 2000. It is a popular sport worldwide, with about 80 million individuals from more than 200 countries participating [10,11]. The number of children and adults participating in martial arts, including TKD, has been increasing by 20–25% annually, and TKD is a popular sport among children [10,11].

Previous studies have reported that regular participation in TKD training can facilitate improvement in body composition by improving aerobic capacity and flexibility and promoting fat loss [12]. In particular, the effectiveness of TKD in improving physical fitness and physical development of children has been reported [11]. Lakes et al. (2013) conducted TKD training in parallel with physical education classes in 600 children in the 7th and 8th grades for 9 months, and suggested that TKD could be effective in improving not only physical fitness, but also cognitive function [13]. In addition, a recent study by Kim et al. showed that participation in TKD training improved children’s brain connectivity from the cerebellum to the parietal and frontal cortex [14].

Thus, it is possible that regular participation in TKD training can improve brain health, e.g., cognitive function, of children during their growth period. The mechanism underlying this effect is unclear, however, and studies on the effects of TKD training on the neurotrophic factors and other growth factors [1,15] involved in neuronal proliferation, migration, survival, differentiation, and synaptic plasticity, and on cerebral blood flow velocity [16], which is implicated in cognitive functions, are limited. Accordingly, the purpose of the present study was to identify the TKD-associated changes in the neuroplasticity-related growth factors in the blood and the cerebral blood flow in healthy children and verify the corresponding changes in their cognitive function.

## 2. Methods

### 2.1. Participants

Subjects in the present study were 30 healthy 4th and 6th grade elementary school students, with 15 students randomly assigned to each of the control (boys, *n *= 9; girls, *n* = 6) and TKD (boys, *n* = 9; girls, *n* = 6) groups. Subjects were not participating in regular exercise other than physical education classes at school, and had no physical or mental illnesses; their physical characteristics are shown in Table 1. The study protocol was approved by the institutional ethics review board of Youngsan University (YSUIRB-201509-BR-002-02). All subjects and their parents were informed about the study procedure and the possible risks involved, and both parents and subjects signed a written consent form.

### 2.2. Anthropometric Measurements

Anthropometric measurements, including height, weight, body mass index (BMI), and maximum oxygen uptake (VO_2_max), were obtained. Height was measured to the nearest centimeter using a stadiometer (SECA213; SECA, Hamburg, Germany). The subjects were instructed to remove their footwear and to stand in an upright position with their feet together. Weight was measured to the nearest 0.1 kg using an electronic balance (H20B; Biospace, Seoul, Korea). Subjects were requested to remove heavy clothing and to stand up straight. The body mass index (BMI) was calculated as the body weight (in kg) without shoes and light clothing, divided by the height (in m), squared. VO_2_max was measured on a motor-driven treadmill (Q65, Quinton, Milwaukee, WI, USA). Expired gases were analyzed using a gas analyzer (Meta Max 3B, Cortex, Wurzburg, Germany). The children performed a modified Balke treadmill protocol [17]. Repeat measurements of height, weight, BMI, and VO_2_max were conducted after 16 weeks of intervention, to record the changes in anthropometric characteristics.

### 2.3. Intervention (TKD Training) Method

The 16-week TKD training protocol was conducted 5 times per week with ratings of perceived exertion (RPE) of 11–15 per training session, each of which lasted 60 min, and was composed of 10 min of basic physical fitness training, 40 min of the main exercise, and 5 min each of warm-up and cool-down exercises involving stretching. The basic physical fitness training involved a shuttle run, Burpee test, and jumping over a person, while the main exercise involved 5 min of basic movements, comprising 6 basic movements and trunk punch in the riding stance, 10 min of Poomsae based on Taegeuk chapters 1–8, 10 min of kicking comprising basic Taekwondo kicks and steps and practice mitt kicking, and 15 min of Taekwon gymnastics. The TKD training method is shown in Table 2.

### 2.4. Blood Collection and Analysis Method

Eight milliliters of blood were collected from the antecubital vein of each subject before and after 16 weeks of intervention, using a 22-gauge needle and a serum separator tube (Becton Dickinson, Franklin Lakes, NJ, USA). Collected blood samples were centrifuged for 15 min at 3000 rpm and then stored at −80 °C until analysis. The analysis of serum BDNF, VEGF, and IGF-1 levels was performed using sandwich enzyme-linked immunosorbent assay (ELISA). For BDNF, we used the human BDNF Quantikine Kit (Catalog No. DBD00; R&D Systems, Minneapolis, MN, USA); for VEGF, we used the human VEGF Quantikine Kit (Catalog No. DVE00; R&D Systems), and for IGF-1, we used the human IGF-1 DuoSet (Catalog No. DY291; R&D Systems). Fluorescence was measured at 450 nm with a microplate reader (Emax; Molecular Devices, Sunnyvale, CA, USA).

### 2.5. Blood Flow Velocity Measurements

Cerebral blood flow velocity was measured twice, before and 16 weeks after the intervention, with reference to a previous study by Aaslid et al. [18]. The peak-systolic (MCAs), end-diastolic (MCAd), and mean cerebral blood flow velocities (MCAm) of the middle cerebral artery (MCA) were measured through the right temporal window, using a 2-MHz pulsed Doppler ultrasound system, with the subject in the supine position, after stabilization for at least 15 min. The Doppler probe was fixed with a headband device to maintain the optimal insonation position and angle. Pulsatility index (PI) was calculated using the following formula: PI = MCAs − MCAd/MCAm.

### 2.6. Cognitive Function Measurements

Cognitive function was measured twice—before and 16 weeks after the intervention—using the Korean version of the children’s version of the Stroop Color and Word Test [19,20] developed by Golden et al. The tests comprised Word reading, Color reading, and Color-Word reading. Each part contained 100 items arranged in five columns and 20 rows. For Word reading, the subjects were asked to read out the three randomly arranged words “RED,” “BLUE,” and “GREEN” written in black ink. For Color reading, the subjects were asked to read out the colors “XXXX” appearing in three different inks. For Color-Word reading, the subjects were asked to read out colors of the words appearing in randomized order. In the present study, the raw scores obtained from subjects reading out loud accurately within 45 s of each condition (Word, Color, and Color-Word) were presented as T scores, which are adjusted scores for age and gender.

### 2.7. Statistical Analysis

Data are expressed as means and standard deviations (SD). All analyses were performed using SPSS version 23.0 for Windows (SPSS Inc., Chicago, IL, USA). Two-way repeated measures analyses of variance (ANOVA) were conducted in order to assess time and group differences in each variable. Both independent and dependent *t*-tests were conducted to examine significant interactions. The level of significance was set at a value of 0.05.

## 3. Results

### 3.1. Changes in Anthropometric Characteristics

Anthropometric characteristics (height, weight, BMI, and VO_2_max) in the control and TKD groups before and after the intervention are shown in Table 3. Two-way repeated measures ANOVA for height, weight, BMI, and VO_2_max interactions between time and group revealed significant differences in VO_2_max (*F* = 7.371, *p* = 0.011) following the intervention. *Post hoc* analysis revealed no significant differences before and after the intervention in the control group, while significant increases in VO_2_max (*p* < 0.05) were observed in the TKD group. However, there were no significant differences in height, weight, and BMI (*p* > 0.05).

### 3.2. Changes in Serum Neuroplasticity-Related Growth Factors

The levels of serum neuroplasticity-related growth factors in the control and TKD groups before and after the intervention are shown in Table 4. Two-way repeated measures ANOVA for serum BDNF, VEGF, and IGF-1 levels, and interactions between time and group, revealed significant differences in serum BDNF (*F* = 9.142, *p* = 0.005), VEGF (*F* = 4.664, *p* = 0.040), and IGF-1 levels (*F* = 4.376, *p* = 0.046) after the intervention. *Post hoc* analysis revealed no significant differences before and after intervention in the control group, while significant increases in serum BDNF, VEGF, and IGF-1 levels (*p* < 0.05) were observed in the TKD group.

### 3.3. Changes in Cerebral Blood Flow Velocities

Cerebral blood flow velocities in the control and TKD groups before and after the intervention are shown in Table 5. Two-way repeated measures ANOVA revealed no significant differences in MCAs, MCAd, MCAm, and PI, or interaction between time and group, after the intervention (*p* > 0.05).

### 3.4. Changes in Cognitive Functions

Cognitive function parameters in the control and TKD groups before and after the intervention are shown in Table 6. Two-way repeated measures ANOVA for Word, Color, and Color-Word tests, and interactions between time and group revealed significant differences in Color-Word test scores (*F* = 13.952, *p* = 0.001) after the intervention. *Post hoc* analysis revealed no significant differences before and after the intervention in the control group, while significant increases in Color-Word test scores (*p* < 0.05) were observed in the TKD group. However, there were no significant differences in Word and Color tests scores (*p* > 0.05).

## 4. Discussion

It has been reported that participation in exercise training, including regular physical activity, in childhood can promote the development of the child’s physical constitution [21], and various diseases such as metabolic syndrome can be prevented through the resultant improvements in body composition and health-related physical fitness [22]. Previous studies have reported that aerobic exercises increase the expression of growth factors such as BDNF, VEGF, and IGF-1, and such growth factors promote the production of neurons [1].

In particular, BDNF is an important nerve growth factor that facilitates the growth and survival of various neurons and regulates synaptic plasticity [23], and IGF-1 has been reported to be an upstream factor in the signaling pathway that regulates BDNF expression [24]. In addition, VEGF is a vascular growth factor and contributes to the production of neurons in the hippocampus [1,25]; interestingly, Palmer et al. (2000) reported that new neurons produced by exercise were mainly observed around the blood vessels [26].

In the present study, the concentrations of serum BDNF, VEGF, and IGF-1 were analyzed to examine the effect of TKD training on neuroplasticity-related growth factors. The levels of BDNF, VEGF, and IGF-1 significantly increased after the training. This suggests that TKD training can induce an increase in neurotrophic factors and growth factors similar to aerobic exercise, suggesting that enhancement of aerobic fitness plays a major role in this process. Fong and Ng reported that TKD training was effective in improving aerobic fitness, in a review of studies on TKD training, while the present study also showed a significant increase in the VO_2_max of the TKD group after the training. Improved aerobic fitness has been reported to be highly correlated with increased IGF-1 levels. Specifically, Whiteman et al. reported that resting serum IGF-1 levels correlated positively with aerobic fitness [27], and increased IGF-1 levels were also related to increased expression of BDNF [24]. Furthermore, Carro et al. showed that injection of IGF-1 into the peripheral blood vessels increased BDNF expression in the hippocampus in animal experiments [24]. Additionally, systemic injection of IGF-1 effectively stimulated angiogenesis in the brain, which was supported by the finding that IGF-1 can induce angiogenesis through VEGF regulation [28].

TCD ultrasonography was developed by Aaslid as a non-invasive method for examining the hemodynamic status of the extra- and intra-cranial blood vessels [18]. Since then, various studies have been conducted on the clinical utility of TCD, and its usefulness for the diagnosis of cerebral vascular disorders in certain conditions, such as traumatic brain injury and cerebrovascular disease, have been reported [29,30]. In addition, this method has recently been used to examine changes in cerebral blood flow under specific conditions, such as hypoxia [31], heat stress [32], and exercise [33], in healthy subjects, and some previous studies have also investigated its relationship with cognitive function [34,35]. The present study measured the MCAs, MCAd, and MCAm of the MCA using TCD, in order to examine the effect of TKD training on the cerebral blood flow velocity, and used these values to calculate the PI. However, we did not find significant changes in MCA, MCAd, MCAm, or PI after TKD training. These results suggest that TKD training interventions did not significantly influence the cerebral blood flow velocity of the MCA. To date, the mechanism underlying exercise-induced cerebral blood flow regulation has not been fully elucidated, but some previous studies have suggested that it is related to the autoregulatory ability to maintain constant blood flow in the brain, despite blood pressure changes and various brain activation demands [36,37].

The autoregulatory mechanism is a concept that includes myogenic regulatory, neurotic, and metabolic factors, and is known to restore cerebral blood flow rapidly when it is decreased due to exercise-induced blood pressure elevation, increased heart rate, sympathetic activation, increased muscle oxygen demand, and reduced oxygen partial pressure (PO_2_). This mechanism functions to meet the overall metabolic demands of the human body [36,38]. That is, it is considered that the cerebral blood flow increases during exercise, as a brain-protective function via the autoregulatory mechanism. Given the above, exercise may improve cerebral blood flow via various effects that can positively influence the autoregulatory mechanism, such as improvement of cardiac ability that may have decreased to subnormal levels, sustained maintenance of blood vessel elasticity, and reduction of blood pressure in the case of metabolic diseases, and reduction of blood cholesterol levels by increased decomposition of body fat. We considered that the cerebral blood flow velocity would not change with exercise, as the subjects in the present study were healthy children without obesity and any specific disease. This assumption was supported by the fact that the subjects in previous studies that reported improvements in the cerebral blood flow velocity through exercise training were suffering from certain diseases, such as stroke [39]. In addition, factors influencing cerebral blood flow velocity include obesity, hypertension, stroke, and type 2 diabetes, and a previous study has reported that high BMI is highly correlated with a reduction in cerebral blood flow velocity and an increase in cerebral vascular resistance [40].

Recent studies have reported that participation in physical activities in childhood may have a positive effect on psychosocial function, including cognitive function and academic achievement [41,42]. The present study used the Stroop Color and Word Test to examine the effect of TKD training on cognitive function. We found a significant increase in the Color-Word Test score, which is a sub-item of the Stroop Color and Word Test, in the TKD group after the training. The result suggests that TKD training can be effective for improving cognitive function, and it is consistent with a previous study [43] that reported a significant increase in the Stroop Color and Word Test scores with regular TKD training. Our results also supported the findings of another previous study [13], which reported that TKD is effective for improving not only physical fitness but also cognitive function. Specifically, Kim reported a significant increase in the Color and Word scores, which indicate changes in cognitive function, after conducting 8 weeks of TKD training in 14 male college students. Similarly, Lakes et al. suggested that TKD is suitable for school physical education classes as an exercise program that improves cognitive function, based on the findings that they obtained after a TKD training intervention composed of traditional TKD techniques (e.g., stances, blocks, strikes, and kicks) and Poomsae (forms) in 600 adolescents [13].

In addition, it appears that the increased BDNF levels may have played a major role in the mechanism by which the TKD training intervention improved the cognitive function in the present study. This point is supported by Cotman et al., who reported that one of the mechanisms underlying the improvement in cognitive function after exercise training was the increased expression of neurotrophic factors, such as BDNF [1].

The present study had several limitations. Since the present study was conducted by a single institution, the number of subjects was small, and it was difficult to clarify whether the changes in all the variables selected in the present study were due to the effects of regular exercise training or the effect of TKD training specifically. In future studies, it will be necessary to verify this aspect through the addition of other exercise groups.

## 5. Conclusions

In conclusion, the results of this study suggest that TKD training may help improve children’s cognitive function, and an increase in the levels of neuroplasticity-related growth factors, such as BDNF, may play a major role in the mechanism underlying this effect.

## Figures and Tables

**Table 1:** Physical characteristics of the subjects at baseline.

Variables/Group	Control (*n* = 15)	TKD (*n* = 15)	*p* Value *
Gender (boys/girls)	9/6	9/6	
Age (years)	11.33 ± 0.72	11.20 ± 0.77	0.630
School grades (unit)	5.33 ± 0.72	5.20 ± 0.77	0.630
Height (cm)	149.00 ± 7.04	148.67 ± 8.19	0.906
Weight (kg)	46.40 ± 6.93	47.60 ± 8.92	0.684
BMI (kg/m^2^)	20.81 ± 1.98	21.58 ± 4.01	0.512
VO_2_max (mL/kg/min)	38.25 ± 6.54	37.89 ± 8.23	0.895

Data are presented as mean ± standard deviation. BMI, body mass index; * Determined using the independent *t*-test.

**Table 2:** TKD training programs.

Procedure	Contents		Time (min)
Warming up	Stretching		5
Main Exercise	Basic physical fitness training	Push-up, Sit-up, Shuttle-run, Burpee test, Vertical jump, Jumping over a person	10
	Basic movement	Close stance, Parallel stance, Riding stance, Forward stance, Forward inflection stance, Backward inflection stance	5
	Poomsae	Underneath blocking, Trunk inner blocking, Trunk punch, Back-fist face front hitting, Elbow turning hitting, Reversed hand knife outward strike, Hand fingertips erect thrusting, Taegeuk chapters 1–8	10
	Kicking	Front kick, Side kick, Round house kick, Downward kick, Step (forward, side, backward), Practice mitt kicking	10
	Gymnastic	Gymnastics composed of TKD movements	15
Cooling down	Stretching		5

**Table 3:** Anthropometric characteristics in the control and TKD groups before and after the intervention.

Variables/Group	Control (*n* = 15)		TKD (*n* = 15)		Time × Group Interaction	
	Baseline	16-Week	Baseline	16-Week	*F*	*p*
Height (cm)	149.00 ± 7.04	150.00 ± 6.90	148.67 ± 8.19	150.00 ± 7.63	0.515	0.479
CV	0.05	0.05	0.06	0.05		
Weight (kg)	46.40 ± 6.93	47.47 ± 7.11	47.60 ± 8.92	47.93 ± 8.02	1.251	0.273
CV	0.15	0.15	0.19	0.17		
BMI (kg/m^2^)	20.81 ± 1.98	21.00 ± 1.94	21.58 ± 4.01	21.35 ± 3.65	2.934	0.098
CV	0.09	0.09	0.19	0.17		
VO_2_max (mL/kg/min)	38.25 ± 6.54	38.17 ± 6.18	37.89 ± 8.23	38.86 ± 7.74 ^#^	7.371	0.011 *
CV	0.17	0.16	0.22	0.20		

Data are presented as mean ± SD. CV, coefficient of variation; TKD, Taekwondo; BMI, body mass index; ^#^ Compared with baseline (*p* < 0.05); * *p* < 0.05.

**Table 4:** The levels of serum neuroplasticity-related growth factors in the control and TKD groups before and after the intervention.

Variables/Group	Control (*n* = 15)		TKD (*n* = 15)		Time × Group Interaction	
	Baseline	16-Week	Baseline	16-Week	*F*	*p*
BDNF (ng/mL)	24.00 ± 7.24	24.45 ± 7.18	24.03 ± 6.16	27.62 ± 7.58 ^#^	9.142	0.005 **
CV	0.30	0.29	0.26	0.27		
VEGF (pg/mL)	179.40 ± 38.63	184.17 ± 31.61	177.54 ± 34.63	193.08 ± 26.19 ^#^	4.664	0.040 *
CV	0.22	0.17	0.20	0.14		
IGF-1 (ng/mL)	377.27 ± 60.77	387.60 ± 60.28	373.96 ± 48.10	402.67 ± 48.48 ^#^	4.376	0.046 *
CV	0.16	0.16	0.13	0.12		

Data are presented as mean ± SD. CV, coefficient of variation; TKD, Taekwondo; BDNF, brain-derived neurotrophic factor; VEGF, vascular endothelial growth factor; IGF-1, insulin-like growth factor-1; ^# ^ Compared with baseline (*p* < 0.05); ** *p* < 0.01; * *p* < 0.05.

**Table 5:** Cerebral blood flow velocities in the control and TKD groups before and after the intervention.

Variables/Group	Control (*n *= 15)		TKD (*n* = 15)		Time × Group Interaction	
	Baseline	16-Week	Baseline	16-Week	*F*	*p*
MCAs (cm/s)	85.20 ± 17.46	86.87 ± 17.27	84.33 ± 21.97	84.20 ± 20.75	1.239	0.275
CV	0.20	0.20	0.26	0.25		
MCAd (cm/s)	45.87 ± 13.11	45.80 ± 12.39	42.00 ± 14.24	42.80 ± 12.30	0.303	0.587
CV	0.29	0.27	0.34	0.29		
MCAm (cm/s)	57.87 ± 14.27	59.33 ± 13.00	55.87 ± 15.28	54.73 ± 13.93	1.063	0.311
CV	0.25	0.22	0.27	0.25		
PI (unit)	0.70 ± 0.13	0.70 ± 0.12	0.77 ± 0.10	0.76 ± 0.09	1.362	0.253
CV	0.19	0.17	0.13	0.11		

Data are presented as mean ± SD. CV, coefficient of variation; TKD, Taekwondo; MCAs, middle cerebral artery systolic blood flow velocity; MCAd, middle cerebral artery diastolic blood flow velocity; MCAm, middle cerebral artery mean blood flow velocity; PI, pulsatility index.

**Table 6:** Cognitive function parameters in the control and TKD groups before and after the intervention.

Variables/Group	Control (*n* = 15)		TKD (*n* = 15)		Time × Group Interaction	
	Baseline	16-Week	Baseline	16-Week	*F*	*p*
Word test (score)	44.67 ± 10.89	45.33 ± 9.06	45.87 ± 8.62	47.60 ± 7.73	1.029	0.319
CV	0.24	0.20	0.19	0.16		
Color test (score)	44.73 ± 8.73	45.27 ± 8.18	46.20 ± 7.40	47.87 ± 6.44	2.429	0.130
CV	0.20	0.18	0.16	0.13		
Color-Word test (score)	49.07 ± 7.84	48.87 ± 8.07	50.33 ± 6.63	52.40 ± 6.23 ^#^	13.952	0.001 **
CV	0.16	0.17	0.13	0.12		

Data are presented as mean ± SD. CV, coefficient of variation; TKD, Taekwondo; ^#^ Compared with baseline (*p* < 0.05); ** *p* < 0.01.
